# Changes in *Bacillus anthracis* CodY regulation under host-specific environmental factor deprived conditions

**DOI:** 10.1186/s12864-016-3004-8

**Published:** 2016-08-17

**Authors:** Se Kye Kim, Kyoung Hwa Jung, Young Gyu Chai

**Affiliations:** 1 Department of Molecular and Life Science, Hanyang University ERICA, 55 Hanyangdaehak-ro, Sangnok-gu, Ansan, Gyeonggi-do 15588 Republic of Korea; 2 Department of Bionanotechnology, Hanyang University, 222 Wangsimni-ro, Seongdong-gu, Seoul 04763 Republic of Korea

**Keywords:** *Bacillus anthracis*, CodY, Environmental signals, Starvation, RNA-sequencing, Chromatin immunoprecipitation

## Abstract

**Background:**

Host-specific environmental factors induce changes in *Bacillus anthracis* gene transcription during infection. A global transcription regulator, CodY, plays a pivotal role in regulating central metabolism, biosynthesis, and virulence in *B. anthracis*. In this study, we utilized RNA-sequencing to assess changes in the transcriptional patterns of CodY-regulated *B. anthracis* genes in response to three conditions of environmental starvation: iron, CO_2_, or glucose deprivation. In addition, we performed chromatin immunoprecipitation on newly identified CodY-mediated genes.

**Results:**

Environmental deprivation induced transcriptional changes in CodY-regulated genes in both wild-type and *codY* null strains, and both CodY-specific and environment-specific patterns were observed. In the iron-depleted condition, overexpression of iron homeostasis genes was observed independent of *codY* deletion; however, transcription of siderophore and amino acid biosynthesis genes was CodY dependent. Although CodY has a significant regulatory role in central metabolism and the carbon overflow pathway, metabolism-associated genes exhibited CodY-independent expression patterns under glucose starvation. Genes that were differentially expressed in response to CO_2_ availability showed CodY-dependent regulation, though their maximal expression did require a supply of CO_2_/bicarbonate.

**Conclusions:**

We speculate that CodY regulates the expression of environmental-responsive genes in a hierarchical manner and is likely associated with other transcription regulators that are specific for a particular environmental change.

**Electronic supplementary material:**

The online version of this article (doi:10.1186/s12864-016-3004-8) contains supplementary material, which is available to authorized users.

## Background

*Bacillus anthracis*, a gram-positive, spore-forming bacterium that is the etiological pathogen of the zoonotic disease anthrax, is constantly exposed to different environmental conditions in its host, strongly influencing its physiology. Several environmental factors are known to promote survival, growth and virulence. One such factor is the presence of carbon dioxide (CO_2_), a gaseous waste product generated by central metabolism and cellular respiration. An infectious pathogen would encounter an elevated level of CO_2_/bicarbonate in infected cells as well as in blood vessels during invasion, switching from aerobic respiration to fermentative growth, adaptation, and virulence induction in various pathogens. Of note, CO_2_-induced virulence factor expression in *B. anthracis* is well documented, as expression of anthrax toxin components and capsule synthesis are enhanced in the presence of bicarbonate and/or high atmospheric CO_2_ levels [1–4].

Glucose availability is also considered an important signal for bacterial pathogens. However, the effect of starvation varies among species. For example, glucose can decrease virulence gene expression in the gram-negative pathogen *Escherichia coli* O157:H7 [5], whereas glucose positively regulates virulence-related processes in *Vibrio cholerae* [6] and *Helicobacter pylori* [7]. As for *B. anthracis*, the presence of glucose increases transcription of anthrax toxin activator *atxA*, in turn positively regulating transcription of protective antigen, one of three anthrax toxin components. This glucose-induced AtxA expression requires the carbon catabolite protein CcpA through an indirect mechanism, forming a molecular link between metabolism and *B. anthracis* pathogenesis [8].

In addition to CO_2_ and glucose, iron availability in the host environment is a major signal for any pathogenic bacterium, as iron is involved in bacterial invasion, survival, motility, capsule biosynthesis and toxin production [9]. To utilize iron in cells or serum, bacterial pathogens express iron-chelating siderophores to scavenge and extract sequestered protein-bound iron ions [10, 11]. As for *B. anthracis*, it expresses the siderophores, petrobactin, and bacillibactin to extract iron in the host system [12], and deletion of iron uptake genes attenuates growth in macrophages and virulence in mice [13]. Overall, host-related environmental factors have a significant impact on bacterial gene expression with regard to stress response, adaptation, survival, and pathogenesis.

Multiple extracellular signals induce complex changes in bacterial gene expression to adapt to new conditions. Simultaneous gene regulation in response to a rapidly changing intracellular milieu suggests the existence of global transcription regulator(s) that integrate extracellular stimuli to choreograph gene transcription. One of the potential gene regulators responding to environmental stimuli is the global transcriptional regulator CodY, which represses early stationary phase genes during growth via direct and indirect mechanism [14]. CodY regulates the expression of the genes closely related to metabolism, adaptation and virulence in various gram-positive pathogens [15]. Its virulence regulation in *B. anthracis* virulence was documented in several literatures [16–18], including a recent CodY overexpression study that showed defects in *B. anthracis* sporulation and pellicle formation [19].

It is notable that CodY-directed gene regulation is closely associated with responses to environmental stimuli. Indeed, nutrient availability is directly associated with CodY binding activity, as starvation leads to depletion of two CodY effectors, GTP and branched-chain amino acids (BCAAs). Without bound effectors, CodY loses its binding affinity and is released from its binding site, followed by derepression of genes involved in various metabolic functions [20, 21]. Previous reports have shown that CodY functions in harmony with CcpA in the regulation of metabolism-related genes. CodY is also known to repress genes that are related to iron uptake and scavenging in *B. anthracis* [17]. However, it remains unclear whether the CO_2_-sensing mechanism is linked to CodY regulation in pathogenic bacteria. At least in *B. anthracis*, post-translational accumulation of AtxA is positively regulated by CodY via unknown mechanisms [18], with its activity also being influenced by CO_2_ [2]. The fact that AtxA is regulated by CodY in a CO_2_ atmosphere suggests potential coordinated regulation between CodY and CO_2_-sensing mechanisms in *B. anthracis*. Although these host-specific environmental stimuli induce stimulus-specific gene expression, it is not yet clear how CodY regulation is connected to environmental-specific responses. Here, we report *B. anthracis* expression profiles in response to both CodY deletion (*codY* mutant) and deficiency of three environmental factors, iron, CO_2_, or glucose, revealing the expression patterns of CodY-mediated genes in *B. anthracis* exposed to various environmental stimuli in a host-like system.

## Results and Discussion

### RNA-sequencing provides additional gene expression patterns in *B. anthracis* Sterne with a *codY* knockout mutation

Transcriptomic profiling using a microarray analysis previously revealed a variety of genes regulated by CodY in *B. anthracis* [18], suggesting its role as a pleotropic regulator that connects regulatory pathways. To further expand the *B. anthracis* CodY regulon and to set a standard for our experiment, we initially isolated total RNA from *B. anthracis* Sterne wild-type (34 F2) and *codY* mutant (BCD22) strains. The cells were cultured in Ristroph medium [22], a defined medium designed to mimic a host-like environment and to maximize anthrax toxin production, and collected during mid-exponential-phase growth. We then performed RNA-sequencing analysis in triplicate using an Illumina HiSeq 2000.

As a result, we identified 251 genes affected by CodY in *B. anthracis* Sterne. Among these genes, 154 up-regulated and 97 down-regulated genes were statistically significant (*q* value < 0.05) in the *codY* mutant strain relative to its parental 34 F2 strain (Additional file 1: Dataset S1). The genes identified in the dataset include both direct and indirect CodY targets, as determined by the presence of previously identified CodY binding motifs [16]. Overexpressed genes in the *codY* mutant strain (i.e., those genes repressed by CodY in 34 F2) are closely related to central metabolism, nucleotide biosynthesis, amino acid biosynthesis, the stress response, and potential virulence, in agreement with previous *B. anthracis* transcriptome profiling studies [18]. Under-expressed genes (positively regulated by CodY) encode tRNA synthases, peptidases, and transporter proteins with various targets; however, due to a large number of hypothetical proteins, their relationships were largely ambiguous. One under-expressed gene is BAS4903, the gene product of which covalently links methionine to its cognate tRNA. In addition, several genes that had previously not been identified in a microarray approach were revealed by RNA-sequencing. For instance, indirect CodY target genes (i.e., genes without a consensus CodY binding motif proximal to their promoter region) include an NLP/P60 family protein (BAS1812) and the tellurium resistance operon *yceCDEF* [BAS0385 (*yceC*), BAS0387 (*yceE*)], the functions of which are suggested to be important to virulence and survival [23, 24]. Overall, the data showed diverse gene targeting by CodY, involving both direct and indirect regulation, and provided new gene sets that are involved in cellular metabolism, survival and virulence in *B. anthracis*.

### RNA-sequencing reveals environmental-specific gene expression patterns for CodY-regulated genes in *B. anthracis* Sterne

**Fig. 1:**
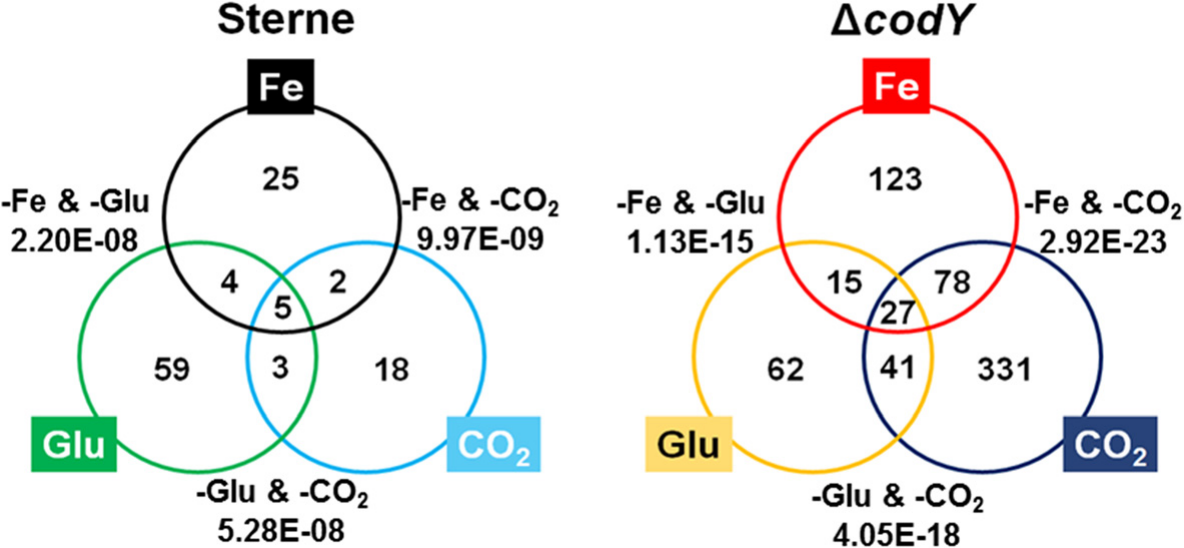
Differentially expressed genes in *B. anthracis* 34 F2 and BCD22 in response to environmental depletion. The Venn diagrams of the genes with statistical significance (*q* < 0.05) in each environmental deprivation treatment (Fe, CO_2_, and Glu) for 34 F2 and BCD strains. The number of differentially expressed genes for each condition, including overlapping genes identified in two or more conditions, is presented in the circles. Statistical significance (*p* values) of overlaps of the Venn diagrams determined by hypergeometric distribution are presented. As for the intersections (i.e., five genes in 34 F2 and 27 genes in BCD), *p* values were much smaller than those of other presented overlaps

Host environmental effects on the survival and virulence of microbial pathogens have been broadly studied and well documented, especially for *B. anthracis*. In this study, we attempted to profile gene sets that respond to the extracellular stimuli readily encountered in the host system, which is a microaerobic environment with limited iron and glucose availability. To identify gene sets in *B. anthracis* that are affected by environmental changes under the host-like condition, we performed RNA-sequencing using RNA libraries prepared from four types of Ristroph media: iron-depleted Ristroph (R^-Fe^), aerated Ristroph (R^-Bic^; i.e., medium prepared without bicarbonate and cultured in a shaking incubator without CO_2_ introduction), glucose-starved Ristroph (R^-Glu^), and a control Ristroph medium. Surprisingly, a small number of differentially expressed genes were identified for the parental strain exposed to environmental deprivation (Additional file 2: Dataset S2). Compared with that grown in the control Ristroph medium (34F2^R^), seven overexpressed and 29 under-expressed genes were observed for the 34 F2 strain grown in R^-Fe^ (34 F2^-Fe^) and nine overexpressed and 19 under-expressed genes in 34 F2 grown in R^-Bic^ (34 F2^-Bic^); in contrast, 32 overexpressed and 39 under-expressed genes were observed for 34 F2 grown in R^-Glu^ (34 F2^-Glu^) (Fig. 1). Few differentially expressed genes were redundantly identified in two [*e.g.*, BAS2111 (34 F2^-Fe^ and 34 F2^-Bic^), BAS4985 (34 F2^-Fe^ and 34 F2^-Glu^) or BAS5333 (34 F2^-Bic^ and 34 F2^-Glu^)] or all of the conditions (*e.g.*, BAS0253, BAS4267, and BAS5155). Approximately one-third of the genes differentially expressed in 34 F2 under starvation encoded hypothetical proteins with unknown domains (34 F2^-Fe^ 7/36; 34 F2^-Bic^ 11/28; 34 F2^-Glu^ 19/71). Two hypothetical proteins found in all conditions were BAS0886 and BAS3872, harboring a sulfite exporter TauE domain and a domain of unknown function, respectively.

Next, to examine the effect of environmental changes on *B. anthracis* CodY regulation, we generated gene profile datasets from the *codY* mutant strain grown in Ristroph, R^-Fe^, R^-Bic^, and R^-Glu^. By comparing differences in expression with BCD22 grown in Ristroph medium (BCD^R^), a large number of genes were differentially expressed in response to starvation (Fig. 1, Additional file 3: Dataset S3); 243 differentially expressed genes were observed in BCD22 grown in R^-Fe^ (BCD^-Fe^) (70 up-regulated; 173 down-regulated), 476 in BCD22 grown in R^-Bic^ (BCD^-Bic^) (283 up-regulated and 193 down-regulated), and 144 in BCD22 grown in R^-Glu^ (BCD^-Glu^) (36 up-regulated and 108 down-regulated). The number of overlapping genes between BCD^-Fe^ and BCD^-Bic^ was 105, that of between BCD^-Fe^ and BCD^-Glu^ was 42, and that of between BCD^-Bic^ and BCD^-Glu^ was 68. Among the overlapping genes, 27 were differentially regulated in all starved conditions. Biological process gene ontology (GO) analysis of genes found in the all three conditions showed enrichment of redox processes and central metabolism (Additional file 4: Dataset S4). Genes down-regulated in BCD^-Fe^ were predicted to contribute to nitrogen metabolism and amino acid biosynthesis, whereas aerobic respiration-related processes were predicted based on genes down-regulated in BCD^-Bic^. The BCD^-Glu^ condition revealed no other biological processes.

We then analyzed gene datasets from a CodY-dependent perspective (Additional file 1: Dataset S1) by comparing RNA-sequencing data between 34 F2 and BCD22 grown under identical conditions (*e.g.*, 34 F2^-Fe^ versus BCD^-Fe^). However, the number of differentially expressed genes was smaller than we first anticipated, with 67 genes in R^-Fe^, 61 in R^-Bic^, and 49 in R^-Glu^ that were differentially expressed by the *codY* knockout strain. Genes that were previously identified to harbor CodY binding sites (*inhA1*, *inhA2*, and *BAS3038*) and that were associated with the deprived molecule (*BAS4424* and *BAS4413*) were found in the analyses, but approximately one-third of the differentially expressed genes were hypothetical proteins (R^-Fe^, 26/67; R^-Bic^, 23/61; R^-Glu^, 17/49). A few hypothetical proteins had putative functions that may contribute to bacterial physiology, such as BAS5235 (prespore-specific transcriptional regulator RsfA-like domain) and BAS0033 (*O*-methyltransferase domain). The small number of differentially expressed genes in the presented analyses may indicate that environment-specific gene regulation is CodY-dependent to a limited degree, and that it involves a few number of genes of unknown functions.

**Fig. 2:**
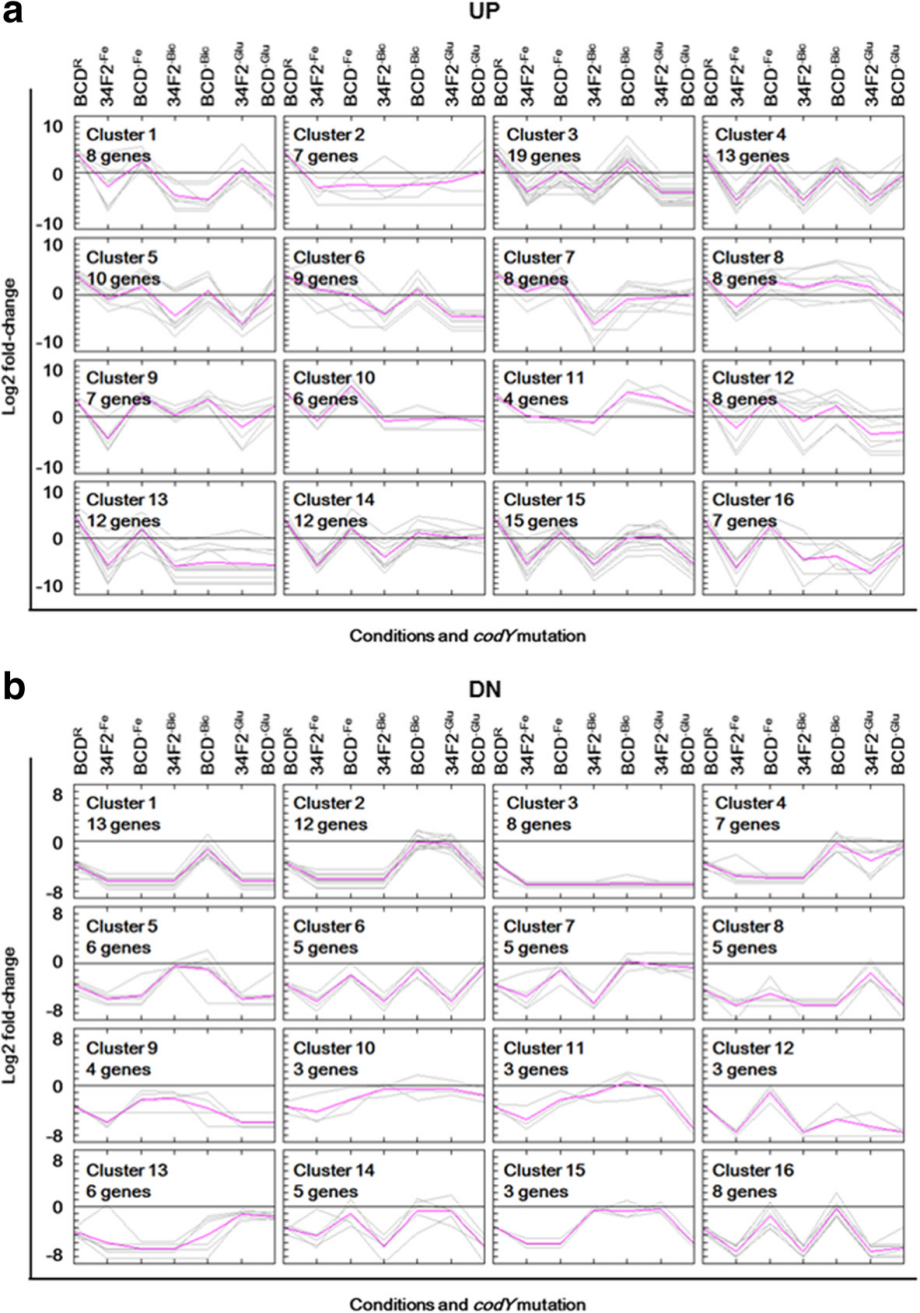
Expressional changes in CodY-mediated *B. anthracis* genes in response to the depleted conditions. The genes negatively regulated by CodY were distributed to 16 clusters (UP and **a**), and the genes positively regulated by CodY were distributed in 16 clusters (DN and **b**), although the variations among the DN clusters were not as dynamic as those of the UP clusters. The log_2_ fold-change values were determined from the means of three independent RNA-sequencing analyses

As CodY was observed to have a reduced regulatory role regarding those condition-specific genes in *B. anthracis*, we focused on the regulatory roles of environmental stimuli on the expression of CodY-regulated genes. To further determine the effect of host environmental factors on transcription patterns in *B. anthracis*, we compared the expression patterns of the CodY-regulated gene set generated from the Ristroph samples with those of the gene sets from three starvation conditions. The fold-change values from eight different gene sets were then subjected to *k*-means clustering to categorize in accordance with their distinct expression patterns, by setting 16 clusters for both negatively and positively regulated genes separately (Fig. 2, Additional file 5: Dataset S5). The number of clusters for *k*-means clustering was determined empirically to classify genes in accordance with their original expression fold-change values as much as possible. The genes positively regulated by CodY were generally repressed under all of the tested conditions, with changes in the degree of repression by derepression due to the *codY* knockout (Fig. 2(a)). The genes negatively regulated by CodY displayed diverse expression patterns in response to environmental changes: some genes were dominantly regulated by at least one environmental stress over the *codY* deletion (clusters 1, 2, 3, 10, 12, 13 and 16), whereas other genes showed CodY-dictated regulation in spite of the depletion of a factor (clusters 4, 5, and 9) or exhibited rather complicated regulation patterns (clusters 6, 7, 8, and 11) (Fig. 2(b)). Taken together, these observations suggest that gene regulation in response to host-specific stimuli and presence of CodY is conditional and/or condition-dependent.

### Validation of RNA-sequencing results using quantitative PCR

To validate gene expression patterns identified in RNA-sequencing and *k*-means clustering, we performed quantitative real-time polymerase chain reaction (qRT-PCR) analyses. Genes to be validated were selected from different clusters; from UP cluster I, we chose the triosephosphate isomerase (*tpiA*; *BAS4987*) and tellurium resistance (*yceC*; *BAS0385*) gene. From DN cluster 4, we chose the methionyl-tRNA synthetase gene (*BAS4903*). From cluster 6, we chose a putative ArsR family transcriptional regulator (*BAS0563*). From UP cluster 7, we chose the phosphopyruvate hydratase gene (*eno*; *BAS4985*). From DN cluster 11, we chose the LysR family transcriptional regulator gene (*BAS5069*). From UP cluster 14, we chose the immune inhibitor A metalloprotease gene (*inhA1*; *BAS1197*). As a result, the qRT-PCR expression profiles of the selected genes were similar to those identified by the RNA-sequencing analysis for conditions under which they were statistically significant, though differences in the extent of the fold change values were observed (Table 1).

**Table 1:** Validation of RNA-sequencing data by qRT-PCR

Target gene	Condition	Fold-change in RNA-sequencing^a^	Fold-change in qRT-PCR
BAS0563	34F2^R^	1	1
Cluster 6	34F2^-Fe^	14.17	402.52
	34F2^-Bic^	ND	1.48
	34F2^-Glu^	ND	1.33
	BCD^R^	6.17	6.69
	BCD^-Fe^	ND	10.44
	BCD^-Bic^	23.50	1.975
	BCD^-Glu^	ND	3.30
*eno* (BAS4985)	34F2^R^	1	1
Cluster 7	34F2^-Fe^	0.13	2.25
	34F2^-Bic^	ND	7.70
	34F2^-Glu^	0.14	0.24
	BCD^R^	7.13	3.25
	BCD^-Fe^	0.97	5.57
	BCD^-Bic^	0.36	0.26
	BCD^-Glu^	0.08	0.37
*tpiA* (BAS4987)	34F2^R^	1	1
Cluster 1	34F2^-Fe^	ND	0.52
	34F2^-Bic^	0.02	4.49
	34F2^-Glu^	0.36	0.04
	BCD^R^	9.85	1.95
	BCD^-Fe^	1.5	1.62
	BCD^-Bic^	ND	0.07
	BCD^-Glu^	ND	0.04
*inhA1* (BAS1197)	34F2^R^	1	1
Cluster 14	34F2^-Fe^	ND	0.87
	34F2^-Bic^	ND	0.43
	34F2^-Glu^	0.25	2.66
	BCD^R^	10.61	37.71
	BCD^-Fe^	1.82	37.56
	BCD^-Bic^	0.43	4.74
	BCD^-Glu^	2.00	20.99
BAS4903	34F2^R^	1	1
Cluster 4	34F2^-Fe^	ND	0.62
	34F2^-Bic^	ND	0.38
	34F2^-Glu^	0.02	0.70
	BCD^R^	0.13	0.15
	BCD^-Fe^	ND	0.12
	BCD^-Bic^	0.37	0.60
	BCD^-Glu^	0.94	0.99
BAS5069	34F2^R^	1	1
Cluster 11	34F2^-Fe^	ND	0.55
	34F2^-Bic^	0.43	0.37
	34F2^-Glu^	0.36	0.75
	BCD^R^	0.14	0.10
	BCD^-Fe^	0.13	0.12
	BCD^-Bic^	3.01	0.90
	BCD^-Glu^	ND	6.33
*yceC* (BAS0385)	34F2^R^	1	1
Cluster 1	34F2^-Fe^	1.35	8.13
	34F2^-Bic^	ND	0.72
	34F2^-Glu^	1.62	0.22
	BCD^R^	13.06	5.68
	BCD^-Fe^	1.26	7.36
	BCD^-Bic^	ND	0.27
	BCD^-Glu^	ND	1.35

^a^ND, not detected in RNA-sequencing

**Fig. 3:**
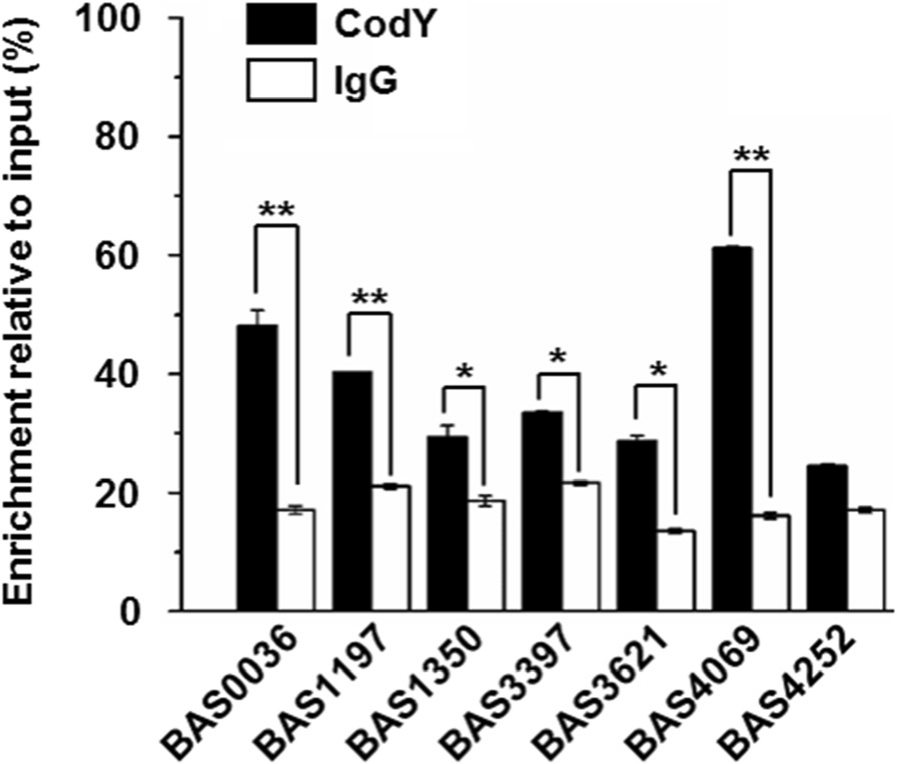
The CodY binding to newly identified CodY-mediated genes in *B. anthracis*. The CodY binding enrichment to its binding motif proximal to the CodY-mediated genes identified in the transcriptome is quantitatively measured by chromatin immunoprecipitation and qRT-PCR. The enrichment of the CodY binding motif is relative to the abundance of the target sequences in the input sample. The values are presented as the means ± SE. *; the level of statistical significance between CodY and IgG enrichment was determined by one-way ANOVA followed by Tukey’s HSD post hoc test (*: *p* < 0.05, **: *p* < 0.01)

In addition to qRT-PCR validation, we selected genes from the RNA-seq datasets and performed chromatin immunoprecipitation quantitative polymerase chain reaction to further validate CodY interaction (ChIP-qPCR) (Fig. 3). Of note, the selected genes were chosen based on the presence of previously defined CodY binding sequence(s) (Table 2) [16]. We observed a 1.5-fold to 3-fold enrichment of CodY at target sequences relative to the negative control (anti-IgG). The binding of CodY to its target sequences varied among the selected genes; the strongest binding to predicted CodY sites was observed for BAS4069 (approximately 3.8-fold enrichment), with BAS4252 showing the weakest (approximately 1.4-fold enrichment). Such variation in CodY enrichment at its motif may represent the differential binding strength of CodY, possibly due to the sequential variation in binding sites compared to the consensus binding sequence, which is similar to what has been previously observed for *B. subtilis* [25]; this assumption, however, requires additional molecular validation. No significant correlation between the binding strength and level of fold change in gene expression was observed. Collectively, we observed differential expression of CodY-mediated genes in response to different environmental signals and confirmed that CodY binds to those genes with CodY binding sequences found in this work.

**Table 2:** CodY binding sites of ChIP-qPCR target genes

Target gene	CodY binding site^a^
BAS0036	AATTTAAAAATTTTTCTGAAAA
BAS1197	ATATCACAATTCAACAATG
BAS1350	ATTGACTAAATTTTCATACAAAACATT
BAS3379	AAAATTAGTAGAATAATAGTTAATAAGAATCTT
BAS3621	CTTAGAAAATTCAGAATGGTAAAATAATATATAAATCGTTGTATGAAAAT
BAS4069	GGAACATACACAGTAAACGATGCGATGTTAGAAGATTTAAAAAATGGTTTTAGTGGTCATCACGCTT
BAS4252	TGAAACTCCCCCTAATAAAAAGTGCCAATATTCCAAA

^a^The presented CodY binding sites were identified by Chateau et al. [16]

### Iron homeostasis and CodY regulation – mutual relationship encompassing amino acid biosynthesis

**Fig. 4:**
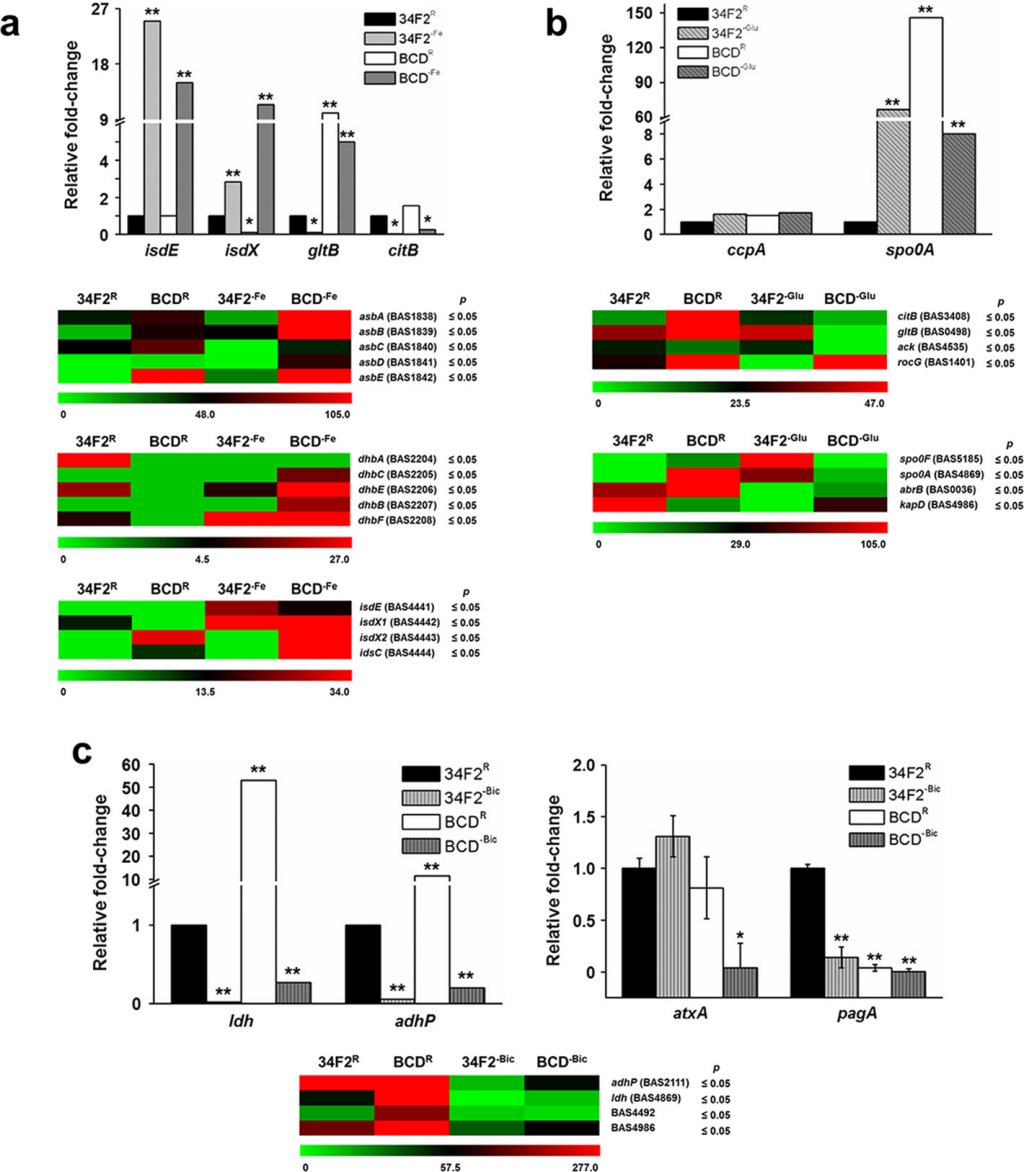
Expressional patterns of pathway-specific genes in response to environmental deprivation. Expressional fold-change values of differentially expressed CodY-mediated genes that were assessed in either **a** iron-depleted condition (R^-Fe^), **b** glucose starved condition (R^-Glu^), or **c** aerated condition (R^-Bic^) are presented in the heat maps and bar graphs. Changes in the mRNA expression are presented in color with green indicating a decrease or red indicating an increase in the read count relative to the parental 34 F2 strain. The term *p* is the statistical significance by the Kruskal-Wallis H nonparametric test for the expression trend of each gene in the wild-type (34 F2) and *codY* null (BCD22) strains under different conditions. Statistical significance in bar graphs were determined by ANOVA followed by Tukey’s HSD post hoc test (*: *p* < 0.05, **: *p* < 0.01)

As mentioned above, iron acquisition is essential for bacterial survival and virulence. Therefore, utilizing iron from the host system is important for the pathogenesis of *B. anthracis*. Strategies bacteria use to do so include degrading iron-bound heme proteins, chelating free iron using siderophores, and taking up iron-bound siderophores. In *B. anthracis*, several genes are involved in iron acquisition and utilization, including petrobactin, bacillibactin, and iron-regulated surface determinant (Isd) genes [12], which are readily overexpressed in iron-depleted medium [26]. Interestingly, overexpression of iron metabolism-related genes (*feoB*, *isdE*, and *isdX*) by iron depletion was observed in the *codY* null strain (Fig. 4(a), Additional file 3: Dataset S3), suggesting that the changes in expression of iron-associated genes occurred in an iron availability-dependent manner rather than as the result of the *codY* mutation. Transcription of iron acquisition and metabolism-associated genes is tightly regulated by the ferric uptake repressor (Fur) protein [27]. Fur represses transcription in the presence of ferrous ions by binding to conserved regulatory motifs (termed Fur boxes) that precede the promoters of iron metabolism genes, thereby affecting virulence and iron homeostasis [28]. Fur boxes are found in Isd genes and several other iron metabolism-associated genes in *B. anthracis* [29, 30], suggesting dominant regulatory control by Fur over its regulon. In addition, according to previous in vitro binding analyses, only two iron-associated genes (BAS1235 and BAS4949) have been identified as direct CodY targets [16], even though *codY* deletion in *B. anthracis* impaired expression of iron uptake genes [17]. Taken together, our findings show that CodY does not directly regulate the expression of iron homeostasis genes, and that these iron homeostasis genes are more sensitive to iron availability than to CodY activity.

Several metabolic processes are iron dependent, including amino acid biosynthesis. Production of glutamate, one of the most abundant amino acids in gram-positive bacteria, is dependent on iron availability as iron is a cofactor of glutamate synthase [31]. In previous studies, transcription of the glutamate synthase genes *gltAB* and aconitase gene *citB* was repressed during iron starvation [32] and also by CodY [33, 34]. We found that the *citB* (*BAS3408*) gene was overexpressed in BCD compared to 34 F2 when grown in both Ristroph and R^-Fe^ media. However, comparison of the fold change values between BCD^R^ and BCD^-Fe^ showed reduced *citB* gene expression by iron depletion. This diminished induction was also observed in the glutamate synthase gene transcription (*BAS0498*) and other metabolism-related genes (Fig. 4(b)). GO biological process analysis of the genes from BCD^-Fe^ compared to those of BCD^R^ showed down-regulation of amino acid biosynthesis. Based on these observations, we speculate that both iron and CodY regulate biomolecule precursor metabolism in a consecutive manner. Derepression of iron-dependent metabolism genes by the *codY* mutation depicts the CodY activity during adaptation to environment with limited iron sources; reduced iron availability would trigger the stringent response as less amino acid products are produced from iron-dependent amino acid biosynthesis. This in turn reduces the binding affinity of CodY to its regulatory sequences, and derepresses amino acid biosynthesis genes, producing a new set of amino acids. Newly synthesized amino acids may then be used for siderophore assembly to overcome iron starvation. Functional analysis of CodY with iron-mediated amino acid biosynthesis using metabolomic approaches would provide a better understanding of the role of CodY in iron starvation.

### Hierarchical regulation between CodY and the glucose-dependent regulator during carbon starvation

Genes that were negatively regulated by CodY from this work displayed dynamic transcription patterns in response to both *codY* mutation and glucose starvation, whereas other sets of differentially expressed genes were mostly repressed by depletion and derepressed by the *codY* mutation (i.e., iron and CO_2_). Notably, transcription patterns shown from genes that fell into UP clusters 2, 3, 6, 7, 10, 12, 13, and 14 were insensitive to the *codY* mutation under glucose starvation (Fig. 2(b)). Biological process analysis predicted that these clusters included glucose metabolism-associated genes, and pathway analyses also showed that these glucose-sensitive genes were involved in pyruvate metabolism and the TCA cycle. However, no significant biological processes were identified in other genes that were derepressed or at least showed diminished repression by the *codY* mutation (UP clusters 4, 5, 9, and 16). Judging from the observed transcriptional patterns, glucose metabolism may be less dependent on CodY regulation during glucose starvation. Indeed, the regulation of central metabolism is not only controlled by CodY but also by other regulators. In particular, CcpA plays a significant role in carbon metabolism, and in many cases, co-recruitment with other regulators determines its activity [35–40]. Transcriptional level of *ccpA* in this work was not significantly affected by glucose starvation nor *codY* deletion (Fig. 4(b)), suggesting that if any changes in CcpA regulon were to occur, it may have been triggered by change in CcpA activity. Comparing the expression patterns in BCD^-Glu^ with BCD^R^ or 34 F2^-Glu^, those genes associated with glucose metabolism and the TCA cycle were differentially expressed in a glucose-dependent manner, masking the regulatory effect of *codY* deletion. Nonetheless, they were differentially expressed in response to *codY* deletion in Ristroph, indicating complexity in regulation of glucose metabolism. Based on these previous observations and our findings, we propose that CodY plays a supportive role in coordinating glucose-dependent central metabolism and other glucose-related processes during starvation in a cooperative manner with CcpA and other glucose-dependent regulators. Interaction between CodY and other regulators, such as CcpA and RpoA, has been documented in *B. subtilis* [41], further supporting our premise of CodY regulation involving additional gene regulators.

Transcription patterns of genes that were categorized in UP clusters 1, 8, 11, and 15 showed repression by the *codY* mutation under glucose starvation. Biological process analysis predicted that these clusters included the monosaccharide metabolic process. Although few genes were shown to be associated with glycolysis/gluconeogenesis, the other genes were related to stress response and cell wall/membrane and also include other vegetative genes. Repression of CodY-mediated genes by *codY* mutation during starvation may be explained by the following scenario: the *codY* deletion may have resulted in failure of the usage of alternative nutrient sources, leading to reduced production of biological precursors and stalled transcriptional machinery. Because glucose is not utilized as a main nutrient source during starvation, *B. anthracis* would switch gene regulation for use of other substances as its carbon source, with CodY playing a pivotal role in the process. Deletion mutation, however, may have caused a regulatory defect in nitrogen metabolism, resulting in failure to utilize amino acids and mimicking a near-complete starvation condition for *B. anthracis*. Lack of carbon sources would suppress any energy-consuming activities, including the transcription of genes required during vegetative growth. Down-regulation of vegetative genes under glucose and nitrogen starvation was observed in *B. licheniformis* [42], though each starved condition was observed separately. Taken together, our findings portray CodY as an essential regulator for coordinating proper transcription of vegetative genes during glucose starvation.

Nutrient starvation induces sporulation. Surprisingly, sporulation regulator genes were down-regulated by the *codY* mutation during glucose starvation (Fig. 4(c)). One of the notable changes was repression of the sporulation regulator *spo0A* gene in BCD^-Glu^. Its transcription was enhanced in both BCD^R^ and 34 F2^-Glu^ (*spo0A* expression in BCD^R^ was greater than that of in 34 F2^-Glu^) compared to 34F2^R^ (expression not detected). However, *spo0A* transcription was repressed in BCD^-Glu^, and its expression level in BCD^-Glu^ was significantly lower than that of in BCD^R^ and 34 F2^-Glu^ (BCD^-Glu^/BCD^R^ = 0.05; BCD^-Glu^/34 F2^-Glu^ = 0.12). This unexpected repression suggests that CodY may act as a positive regulator for *spo0A* transcription in the mid-exponential growth phase during starvation. A positive effect of CodY on *spo0A* expression was previously identified in *B. subtilis* [43], which suggested a complex regulatory pathway in Spo0A-mediated sporulation during carbon starvation, contradicting a previous model where CodY represses transcription of sensor kinase *kinB* and extracellular Phr peptides (*phrA* and *phrE*) that positively regulate sporulation [34, 44, 45]. As for this study, transcription of *kinB* and Phr peptides was not detected. One possible explanation for CodY as a positive regulator for *spo0A* transcription is that there may be CodY-regulated sporulation inhibitors that repress *spo0A* transcription. This assumption, however, requires identification and functional characterization of such inhibitors.

### Intervention of potential CO_2_-dependent regulators at CodY-mediated CO_2_-responsive genes

Genes that were differentially expressed in response to CO_2_ deprivation can be categorized into two classes: (i) CodY-mediated genes that were repressed by CO_2_ deprivation and derepressed by *codY* mutation, and (ii) genes that were not affected by the mutation. The genes that were derepressed by *codY* deletion (i.e., genes from UP clusters 3, 4, 5, 6, 11, 12, 14, and 15) were involved in amino acid catabolism and ABC transporter system, but their expression levels in BCD^-Bic^ were lower than those of in BCD^R^. The expression patterns in this study coincided with a comparative transcriptome profiling of *B. cereus* strains under CO_2_ or aerobic atmospheres [46]. Our findings suggest that CodY acts as a repressor during aerobic growth and that transcription of CodY regulon is also affected by the presence of CO_2_/bicarbonate. One of the interesting findings is that the CO_2_-depleted transcriptome showed diminished derepression of CodY-mediated genes. One instance is the diminished transcription of the immune inhibitor A metalloprotease (*inhA1*) gene, one of many secreted *B. anthracis* proteases that targets various substrates contributing to virulence [47] or benefiting cell survival [48]. Transcription of *inhA1* occurs under both air and in toxin-inducing conditions (*i.e.*, 5 % CO_2_ and bicarbonate), with the latter condition being more favorable for higher expression [49]. It is also suggested that *inhA1* is a direct target of CodY [16]. Our transcriptome profiling, qRT-PCR and ChIP-qPCR data showed overexpression of *inhA1* in the BCD strain and enrichment of CodY at its binding site proximal to the *inhA1* ORF (Fig. 3 and Table 1), confirming previous observations. Interestingly, *inhA1* overexpression in BCD^R^ was significantly diminished in BCD^-Bic^, suggesting that additional CO_2_-related regulators are required for full *inhA1* expression. This raises the possibility of direct/indirect interplay between CO_2_-dependent regulators and CodY. Examples of such CO_2_-dependent regulators are the aerobic/anaerobic two-component system ResD-ResE in *B. subtilis* [50, 51] and the homologue system BrrA-BrrB in *B. anthracis* [52]. Interaction of these systems with CodY requires further investigation, which is required to fully elucidate the underlying mechanism.

The genes insensitive to *codY* mutation (clusters 1, 2, 7, 8, 9, 10, 13, and 16) were predicted to be involved in glycolysis and fermentation (*e.g.*, BAS2111, BAS4492, BAS4869, BAS4986, BAS4987, and BAS4989) (Fig. 4(c)). This observation suggests that transition from aerobic to anaerobic respiration is indirectly mediated by CodY, at least in terms of glycolysis and fermentation-related gene transcription. Regulation of anaerobic fermentation genes is well documented in *B. subtilis*, as the extracellular CO_2_/bicarbonate level is relayed via a regulatory cascade that involves ResDE and the anaerobic transcription regulator Fnr, which in turn activates expression of lactate dehydrogenase and alcohol dehydrogenase [53]. As no CodY binding sites proximal to anaerobic fermentation genes were found nor was any solid regulatory connection between CodY and CO_2_/bicarbonate established, our findings show no direct involvement of CodY in transcription of anaerobic metabolism-related genes during the aerobic-anaerobic transition.

One of CO_2_-mediated regulatory pathways in *B. anthracis* includes virulence expression regulation by the anthrax toxin activator AtxA. Although its interaction with CO_2_ is yet unclear, it was previously suggested that elevation in the level of CO_2_/bicarbonate enhances multimerization of AtxA, and that the AtxA multimerization is closely associated with its protein function [2]. In this work, reduced induction of anthrax toxin component protective antigen was observed in all perturbed conditions (i.e., 34 F2^-Bic^, BCD^R^ and BCD^-Bic^) (Fig. 4(c)). Transcriptional level of *atxA* gene was somewhat changed in 34 F2^-Bic^ and BCD^R^ but was within the error range, suggesting that the sole effect of CO_2_/bicarbonate or *codY* deletion on *atxA* transcription is minor. On the contrary, *atxA* transcription was significantly depressed in BCD^-Bic^ (Fig. 4(c)). Although the underlying mechanism is unclear, we are tempting to explain the presented result as follows: CodY may be a member of gene regulatory complex for *atxA* transcription along with other CO_2_-responsive regulators (such as the two-component system BrrA-BrrB [52]), as shown previously that there is a CodY binding site proximal to *atxA* promoter [16], but the binding affinity is weak and its effect on the transcription is minor. Instead, it may cause DNA curvature that would repress transcription but induce recruitment of regulatory complex, allowing *atxA* to be transcriptionally “poised”. Bicarbonate deprivation or *codY* deletion may reduce *atxA* transcription, but both conditions can compensate each other to sustain *atxA* transcription. As for the BCD^-Bic^ condition, synergic regulation of CodY and CO_2_-responsive regulators on the *atxA* promoter is lost, ultimately leading to depression of *atxA* and anthrax toxin gene transcription. Further characterization and validation of CO_2_-responsive regulators with CodY may reveal the regulatory mechanism between CO_2_/bicarbonate, nutrition and virulence expression.

## Conclusions

**Fig. 5:**
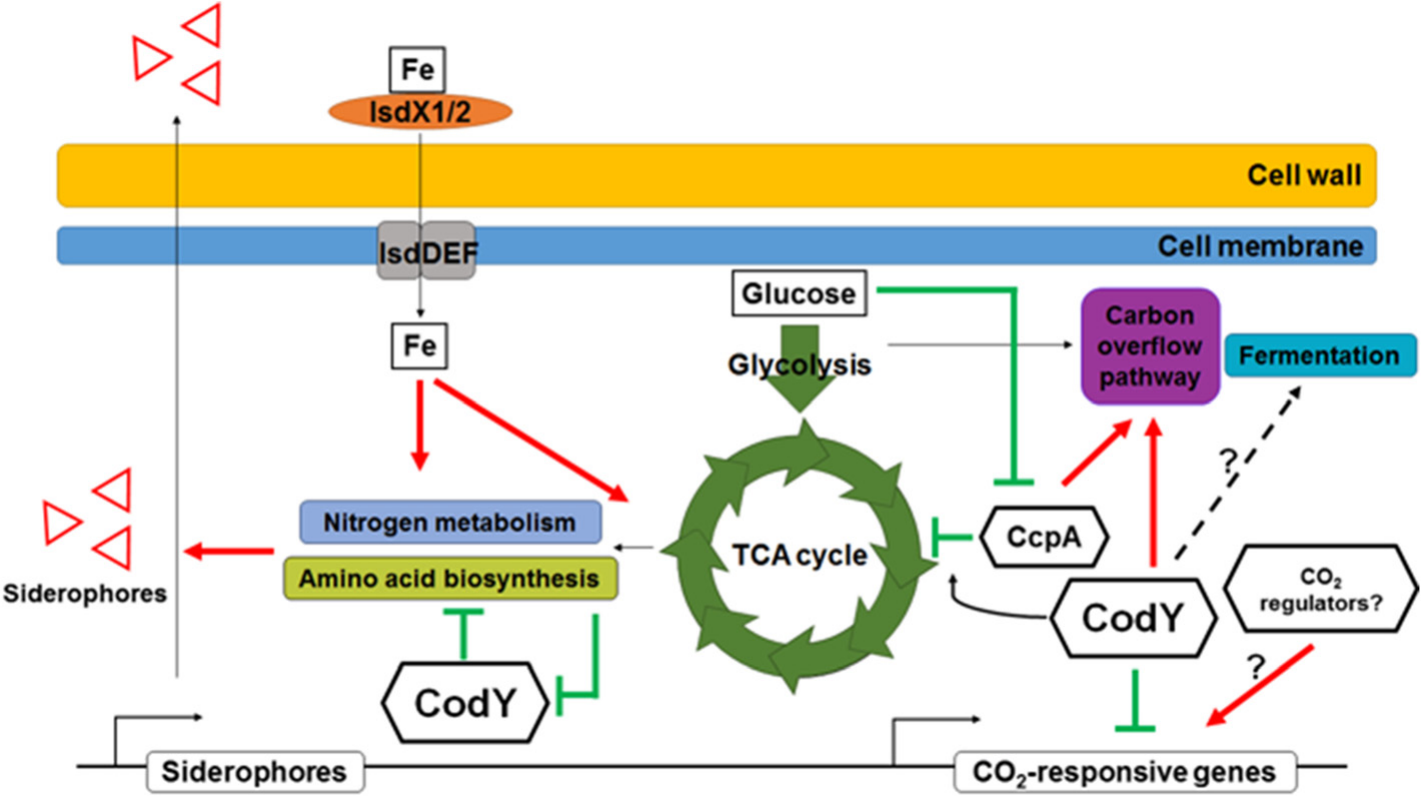
A schematic illustration of the proposed CodY regulation in *B. anthracis* in response to environmental factors

We attempted to determine the expression patterns of *B. anthracis* genes that are directly or indirectly regulated by CodY in response to different environmental deprivation conditions using high-throughput sequencing. We identified CodY-mediated genes that were either sensitive or insensitive to environmental changes, with some being involved in regulation under more than one starvation condition. Although transcriptional repression in response to environmental factor deprivation was derepressed by *codY* mutation in several cases, the CodY-mediated genes displayed complex expression patterns, being either positively or negatively regulated in response to the mutation. The genes with complex transcription patterns were closely related to specific biological pathways that utilize the deprived environmental factor(s) for proper regulation and metabolism. These biological pathways were regulated by multiple regulators that may act in a global or a pathway-specific manner, such as the Fur regulon for iron metabolism and CcpA for glucose metabolism. Our transcription profiles suggest that CodY regulation is sophisticatedly coordinated with other regulators during the environmental changes evaluated (Fig. 5): (i) iron metabolism is regulated by iron-dependent regulators, is independent of CodY repression but affects CodY-mediated regulation indirectly by altering iron-dependent amino acid biosynthesis; (ii) CodY assists other glucose-dependent regulators in the regulation of glucose catabolism and the TCA cycle, and indirectly coordinates proper transcription of vegetative genes in response to glucose availability; and (iii) CodY retains its role as a global regulator for those CodY-mediated genes that require CO_2_/bicarbonate for full expression but has less of a regulatory role in aerobic-anaerobic respiration, at least in enzyme gene regulation. The complex nature of CodY regulation in response to environmental factors suggests the involvement of other pleiotropic or pathway-specific regulators. As numerous gene regulators and regulatory proteins belong to the CodY regulon [54], functional characterization of CodY-mediated gene regulators would elucidate this complex phenotypic changes. Our findings also depict CodY as a supportive regulator rather than a signal-integrating hub in response to the deprived conditions studied. This transcription study provides useful insight into CodY regulon in *B. anthracis* during various environmental changes that would ultimately affect its physiology and virulence. However, we only examined a limited set of environmental stimuli that *B. anthracis* experiences during its life cycle inside or outside of the host system. Furthermore, interaction of CodY with other regulators requires validation of their direct physical contact or indirect regulation during transcriptional regulation in the host-like environmental niche. Other environmental factors, such as amino acid starvation, are being studied by many research groups [55]. Integration of datasets generated using environmental factors that affect other physiological and virulence features of *B. anthracis* would provide better information for understanding its adaptation and pathogenesis in the host system.

## Methods

### Bacterial strains and media conditions

Strains and vectors used in this study are listed in Table 3. *B. anthracis* Sterne 34 F2 was used as the parental strain for this experiment. The *codY* knockout mutant BCD22 was constructed using an integration vector pKS1, which was kindly provided by Dr. Konstantin Shatalin using the method previously described [56]. In brief, a gene fragment containing 500 bp upstream of *codY* and another fragment containing 500 bp downstream of *codY* were amplified with primer sets 500CodYUP and 500CodYDN, respectively. The upstream fragment was inserted into pKS1 that had been digested with *Eag*I and *Pst*I, and then the downstream fragment was inserted after digesting with *Hind*III and *Kpn*I. The constructed plasmid was designated as pKSCodY. After removing methylation by transforming the plasmid into *E. coli* ER2925, pKSCodY was introduced into *B. anthracis* Sterne as described by Koehler’s group [3]. Replacement of the *codY* open reading frame was performed according to the method described previously with modifications as follows: transformed cells displaying both kanamycin (Km) and erythromycin (Em) resistance were selected and grown in LB + Em at 42 °C (non-permissive) for 5 generations. Cells resistant to Km and Em were selected and grown in LB without antibiotics at 30 °C (permissive) for approximately 5–10 generations. The cultures were diluted and spread on LB + Km plates and incubated at 37 °C. The cells that lost Em resistance were identified by replica plating. The Em-susceptible colonies were selected to extract the genomic DNA, and the loss of *codY* was confirmed by sequencing and immunoblotting using anti-CodY antibody (Additional file 6: Figure S1).

**Table 3:** Strains and plasmids used in this study

Bacterial strains	Description^a^	Source or reference
*B. anthracis* strains		
34 F2	Wild-type Sterne; pXO1^+^, pXO2^−^	Laboratory stock
BCD	34 F2 Δ*codY*::*aphA3*, Km^R^	This study
*E. coli* strains		
DH5α	*supE44* Δ*lac U169 (*φ*80lacZ*Δ*M15) hsdR17 recA1 endA1 gyrA96 thi-1 relA1*	New England Biolabs
ER2925	*ara-14 leuB6 fhuA31 lacY1 tsx78 glnV44 galK2 galT22 mcrA dcm-6 hisG4 rfbD1 R(zgb210::Tn10)TetS endA1 rpsL136 dam13::Tn9 xylA-5 mtl-1 thi-1 mcrB1 hsdR2*	New England Biolabs
Plasmids		
pKS1	An integration vector designed for efficient gene inactivation in *B. anthracis*; Km^R^, Em^R^	[56]
pBCD22	pKS1 derivative harboring 500 bp fragment upstream and 500 bp fragment downstream of *codY* flanking *aph3A*; Km^R^, Em^R^	This study

^a^Km^R^, kanamycin resistant; Em^R^, erythromycin resistant

The Ristroph medium was used as a host-mimicking and toxin-inducing medium for this experiment. Cells were initially grown overnight in brain heart infusion (BHI) medium, supplemented with Km when growing knockout mutant cells, and inoculated to Ristroph media (initial OD_600_ = 0.05). Unless stated otherwise, all cells were inoculated in the Ristroph medium and grown at 37 °C under 5 % CO_2_ atmosphere with shaking (150 rpm). In this experiment three environmental factors were manipulated to observe the effect of environmental factors on *B. anthracis* gene expression: CO_2_, iron and glucose. For iron depletion stress, 450 μM of 2,2’-dipyridyl (Sigma-Aldrich, St. Louis, MO, USA) was added to the Ristroph medium prior to inoculation. For air/CO_2_ differential gene expression analysis, sodium bicarbonate was omitted in the process of preparing the Ristroph medium, and cells were grown with aeration in a shaking incubator without CO_2_. For glucose starved samples, glucose was omitted in the process of preparing the Ristroph medium. Cells were harvested at the mid-exponential phase (OD_600_ = 0.5 – 0.6), washed with PBS, and frozen at −80 °C until further use.

### RNA extraction, library preparation and RNA-sequencing

The total RNA samples from the collected cells were extracted using the Qiagen RNeasy Mini Kit in accordance with the manufacturer’s instructions. Prior to library preparation, ribosomal RNAs from the total RNA samples were depleted using a RiboMinus Bacteria/Yeast Transcriptome Isolation Kit. The library construction for Illumina HiSeq sequencing was performed using the NEBNext® Ultra™ Directional RNA Library Prep Kit for Illumina® and according to the manufacturer’s instructions. Pair-end sequencing on Illumina HiSeq2000 was performed at Macrogen (http://www.macrogen.com/kor/) in triplicate.

### Data analysis

The expression profiling of the *B. anthracis* parental and mutant strains was determined using Rockhopper version 1.30 according to the authors’ instructions (for more information, refer to [57]). Reads were aligned to a reference genome (*B. anthracis* str. Sterne, NC_005945.1), following the process similar to Bowtie2 [58]. Quality of read alignment was controlled by Phred score threshold [59, 60]. Rockhopper normalized read counts by the upper quartile gene expression level after excluding genes with zero expression [61]. The genes were considered differentially expressed if the *q*-value (determined using the Benjamini-Hochberg procedure [62]) was equal to or below 0.05 (*q* < 0.05) and if the log_2_-fold-change was greater than 1 or less than −1. For Venn diagram, significances of overlapped genes were determined by hypergeometric distribution test.

Differentially expressed genes from the dataset were functionally annotated using the Database for Annotation, Visualization and Integrated Discovery (DAVID) informatics tool version 6.7 [63]. For GO Term analysis we used the GO FAT default setting (“*Bacillus anthracis*” as the provided background). Fold enrichment values and statistical significance values (i.e., *p* values, Bonferroni, Benjamini, and FDR) were calculated by DAVID software. The significances of GO terms were determined using *p* values.

Clustering analyses were performed in MultiExperiment Viewer (MeV, version 4.7.3) using the 34F2^R^ vs BCD^R^ gene set [64]. K-means clustering were performed using Pearson correlation distance. Significance between conditions were assessed using Kruskal-Wallis H nonparametric test.

### Quantitative PCR and CodY ChIP-qPCR

All primers used in this study are listed in Table 4. To validate the expressional patterns identified from the RNA-sequencing data, quantitative real-time PCR (qRT-PCR) was performed with cDNA templates synthesized from previously extracted RNA samples, SYBR® Premix Ex *Taq*™ II (TaKaRa BIO) and the appropriate primers using the ABI 7500 Real-Time PCR system (Applied Biosystems, Carlsbad, CA, USA), as follows: First-strand cDNA templates were synthesized from extracted RNA samples using SuperScript II Reverse Transcriptase (Invitrogen) following the manufacturer’s instructions. Quantitative real-time PCR (qRT-PCR) was performed as follows: 10 μl of SYBR® Premix Ex *Taq*™ II, 0.4 μl of ROX, 4 pmol of forward primer, 4 pmol of reverse primer, and 1 μg of cDNA were added with water to a final volume of 20 μl. The mixture was amplified for 40 cycles with an initial melt at 95 °C for 15 s and 60 °C for 1 min. The threshold cycle (C_t_) of each gene was normalized to that of the housekeeping gene *gyrB* [65]. The relative expression differences were calculated using the 2^-ΔΔCt^ method [66].

**Table 4:** Primers used in this study

Oligonucleotide^a^	Sequence^b^ (5’ → 3’)	
	Forward	Reverse
500CodYUP	ATA**GGATCC**GAATTATTAGCAAAAAC	ATA**TCTAGA**TTAGTTTGTTTTTAATTTAGCA
500CodYDN	GGG**CGGCCG**CGTATCACGTGAAGG TGTGC	CAC**CTGCAG**TGGAAACTAGGGCGA GTCAC
BAS0563	ACACCACAAGAGACATGTTCT	CGCATGTAATATTCTCGTACGTGT
*yceC* (BAS0385)	TCGATTTAACGAAAGGACAACCA	ACAACGGATGCATCACAATCT
*inhA1* (BAS1197)	CATCAATCGCTTTGACAGCTGT	GCGCATCTGCTAAACGTTCT
BAS4903	GGCCGTATGCAAATGGTTCG	GCCCGAATTGCAATTGGTGT
*eno* (BAS4985)	TGTTTATGCTCGCGAAGTCCT	TGCTTCGTGCTCACCAGTAG
*tpiA* (BAS4987)	GCGTAAACCAATTATCGCAGGT	ACCAGGCGCTCTAAGAATAGA
BAS5069	TCGCAAGATTAGAGCATATGACA	GCCCTTGGCGGTCAAATAAC
BAS0036ChIP	ATTTTTAAATTGTAAGTGGAGATTGT	ACCATCTATTACTCATATTTCAAGAA
BAS1197ChIP	TTCAGAAAACATTGTTGAATTGTG	ATTTGTAAAAATTCCATAGTTAGCAT
BAS1350ChIP	AGCACAAATGATACTTGCAAA	TTAACGACATGTGTTGGACT
BAS3397ChIP	ATTTTTCGAGTTTTGTAAAGTGTT	TCAATGAGGTATAGTGTTTTAAAAGA
BAS3621ChIP	AGTTAATGAACATGCAATTTTCAT	GTAGTTTTATTTACTTAGAAAATTCAGAAT
BAS4069ChIP	ATCGCATCGTTTACTGTGTA	GGATTACTTCGTCCAATCGA
BAS4252ChIP	TAACTTTTTGGAATATTGGCACT	TTTTCATTGAAACTCCCCCT
BAS4963ChIP	AGCTCTCTGGAAAGAATGTG	CGTAAAAGCGTTGAAAAGGA

^a^ChIP, chromatin immunoprecipitation; denotes primers that were used in ChIP-qPCR

^b^Boldface bases denote a restriction enzyme target site

To validate the CodY binding to the identified genes, chromatin immunoprecipitation quantitative PCR (ChIP-qPCR) was performed. Primers for ChIP-qPCR were designed based on the CodY binding sequences identified from a previous in vitro CodY binding analysis [16]. Immunoprecipitation of CodY-bound DNA fragments was performed as follows: 34 F2 cells were inoculated in 400 ml Ristroph medium and grown at 37 °C under 5 % CO_2_ atmosphere until reaching mid-exponential absorbance. Formaldehyde was then added for crosslinking to final concentration of 1 %, and incubated at 25 °C for 30 min. Glycine was added to quench the reaction (final concentration of 125 mM), and cells were harvested by centrifugation at 14,000 g for 30 min. After washing with PBS, cells were resuspended in 1.5 ml sucrose-malate-magnesium buffer (20 mM maleic acid, 500 mM sucrose, 20 mM MgCl_2_) supplemented with 1 mg/ml lysozyme and 1 mM PMSF, and incubated at 37 °C for 1 h. Cells were then collected by centrifugation at 6000 g for 5 min at 4 °C, and resuspended in 0.5 ml Brij buffer [100 mM Tris-Cl, 200 mM NaCl, 1 % (v/v) Triton X-100, 0.1 % (w/v) sodium deoxycholate, 20 % (w/v) glycerol, 0.2 % (w/v) Brij 58 (Sigma-Aldrich), pH 7.5]. Resuspended cells were treated with 10 μl RNase A (10 mg/ml), 50 μl Mg-Ca buffer (100 mM MgCl_2_, 50 mM CaCl_2_), and 1 U DNase I (New England Biolabs, Ipswich, MA), and incubated at 37 °C for 1 h with shaking. DNA fragmentation was terminated by adding 1.5 ml Solution A [20 % (w/v) sucrose, 50 mM NaCl, 10 mM EDTA, 10 mM Tris, pH 8.0]. Cells were then sonicated using Diagenode Bioruptor® Plus Sonication Device to fragment genomic DNA to size of 70 – 200 bp (high; 10 s ON, 10 s OFF cycle for 15 min). Samples were centrifuged at 14,000 g for 15 min to remove any debris. Fragmented DNA samples were then immunoprecipitated using a method described previously [67] with modifications of using anti-CodY antibody as a primary target antibody and washing with Solution A buffer for five times. Immunoprecipitated DNA fragments were de-crosslinked by heating samples at 65 °C for 16 h, and purified using phenol-chloroform purification. Purified DNA samples were used to perform qPCR, using the aforementioned qRT-PCR method with modifications of using no less than 0.5 ng DNA per reaction instead of 1 μg cDNA, and annealing and elongation step temperature of 55 °C instead of 60 °C.

## Additional files

Additional file 1: Dataset S1. Comparison of differentially expressed genes between *B. anthracis* Sterne 34 F2 and *codY*-deleted strain BCD22 in various deprived conditions. (XLSX 44 kb) Additional file 2: Dataset S2. Differentially expressed genes of *B. anthracis* Sterne 34 F2 in various deprived conditions. (XLSX 23 kb) Additional file 3: Dataset S3. Differentially expressed genes of *codY*-deleted *B. anthracis* Sterne BCD22 in various deprived conditions. (XLSX 74 kb) Additional file 4: Dataset S4. Gene ontology analysis of differentially expressed genes in *codY*-deleted *B. anthracis* Sterne BCD22 in various deprived conditions (XLSX 17 kb) Additional file 5: Dataset S5. *k*-means clustering of CodY targets in various deprived conditions. (XLSX 14 kb) Additional file 6: Figure S1. Validation of the *codY* deletion in *B. anthracis* BCD22. Immunoblotting of protein samples from a control 34 F2, *codY*-deleted BCD22, and *codY*-complemented BCC7 against anti-CodY antibody. Equal amount of protein (10 μg) was used for each sample. (TIF 87 kb)

## Abbreviations

BCAA: Branched-chain Amino Acid
BHI: Brain Heart Infusion
ChIP-qPCR: Chromatin Immunoprecipitation Quantitative Polymerase Chain Reaction
CO_2_: Carbon Dioxide
DAVID: the Database for Annotation, Visualization and Integrated Discovery
EDTA: Ethylenediaminetetraacetic Acid
Em: Erythromycin
GO: Gene Ontology
GTP: Guanosine Triphosphate
Km: Kanamycin
MeV: MultiExperiment Viewer
PBS: Phosphate Buffered Saline
PMSF: Phenylmethylsulfonyl Fluoride
qRT-PCR: Quantitative Real-time Polymerase Chain Reaction
