# Supplementary figures and images for: Changes in Bacillus anthracis CodY regulation under host-specific environmental factor deprived conditions
Source: BMC Genomics. 2016 Aug 17;17:645. doi: 10.1186/s12864-016-3004-8 (PMC4987991; doi:10.1186/s12864-016-3004-8)

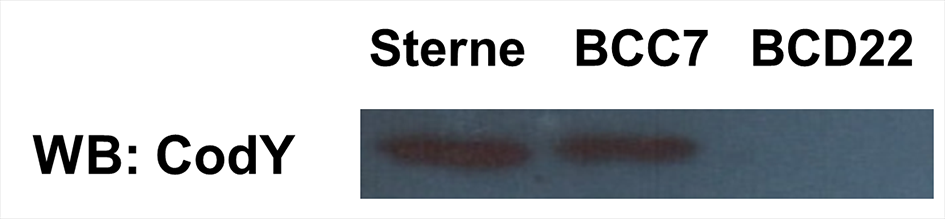

Supplement: Additional file 6: Figure S1. — Validation of the codY deletion in B. anthracis BCD22. Immunoblotting of protein samples from a control 34 F2, codY-deleted BCD22, and codY-complemented BCC7 against anti-CodY antibody. Equal amount of protein (10 μg) was used for each sample. (TIF 87 kb) [file 12864_2016_3004_MOESM6_ESM.tif]
